# Understanding Patchy Landscape Dynamics: Towards a Landscape Language

**DOI:** 10.1371/journal.pone.0046064

**Published:** 2012-09-25

**Authors:** Cédric Gaucherel, Frédéric Boudon, Thomas Houet, Mathieu Castets, Christophe Godin

**Affiliations:** 1 UMR AMAP - INRA, Montpellier, France; 2 French Institute of Pondicherry, IFP - CNRS, Pondicherry, India; 3 INRIA – DAP, CIRAD, Montpellier, France; 4 GEODE UMR 5602 CNRS – Université Toulouse 2 Le Mirail, Toulouse, France; UGent/VIB, Belgium

## Abstract

Patchy landscapes driven by human decisions and/or natural forces are still a challenge to be understood and modelled. No attempt has been made up to now to describe them by a coherent framework and to formalize landscape changing rules. Overcoming this lacuna was our first objective here, and this was largely based on the notion of Rewriting Systems, also called Formal Grammars. We used complicated scenarios of agricultural dynamics to model landscapes and to write their corresponding driving rule equations. Our second objective was to illustrate the relevance of this landscape language concept for landscape modelling through various grassland managements, with the final aim to assess their respective impacts on biological conservation. For this purpose, we made the assumptions that a higher grassland appearance frequency and higher land cover connectivity are favourable to species conservation. Ecological results revealed that dairy and beef livestock production systems are more favourable to wild species than is hog farming, although in different ways. Methodological results allowed us to efficiently model and formalize these landscape dynamics. This study demonstrates the applicability of the Rewriting System framework to the modelling of agricultural landscapes and, hopefully, to other patchy landscapes. The newly defined grammar is able to explain changes that are neither necessarily local nor Markovian, and opens a way to analytical modelling of landscape dynamics.

## Introduction

Understanding the emergence of landscape patterns is still a challenge. It is sometimes convenient to classify landscapes into two different types, the relatively continuous mosaics such as colonization patterns, and the so-called patchy landscapes such as agricultural and other anthropogenic mosaics. While the former have begun to be well understood, with help from various diffusion or dispersion mechanisms [1], the dynamics of the latter are only poorly understood [2], [3]. In particular, it is not possible to find any mathematical formalization of patchy landscape dynamics. Patchy landscapes are assumed to be composed of several units (or patches), supposed to be uniform and relatively autonomous, showing sharp boundaries with their neighbours. Such landscape units are highly dependent on the landscape’s topology (i.e. the neighbouring relationships, possibly non-local [2], [4], [5]), and cannot be handled as grid-based (with pixels) mosaics [6], [7]. There is a great deal of misunderstanding in this regard, and it is crucial to understand that we are discussing in this study the landscape mosaic (visible landscape units) rather than the ecological processes (colonization or other spatial spread) supported by the mosaic itself. We propose here, in what may well be the first attempt ever made, to formalize patchy landscape dynamics.

We meet with a large number of difficulties in the modelling of patchy landscapes [8]. A landscape is a spatial object usually exhibiting a non-stationary property: its statistical moments computed on subparts of it are not constant. This is the obvious consequence of any discontinuity (a boundary, also called a singularity in the terminology of differential equations) between its units. In addition, a landscape is a temporal object exhibiting non-Markovian (non-stationary) dynamics, i.e. with changes independent of previous landscape states [9]. Anthropogenic mosaics are an obvious pitfall, as human decisions driving spatial and dynamic patterns are numerous, of various natures and interacting at various scales simultaneously [2], [3]. These difficulties explain why, in the early modelling of landscapes, most models started with grid-based structures and Markovian-like dynamics. To do so, they simplified landscape changes into less realistic cellular automata or individual-based models. Up to now, landscapes have been modelled using mechanistic [6], [10] as well as statistical rules [9], [11]. In this context, particularly revealing is the observation that no concrete attempt has yet been made to define a coherent modelling framework, such as a mathematical description to formalize landscape-changing rules. The aim of this study is not to give the detailed mathematical treatment of the formalization, but to explore the relevance of such formalization.

Many other fields of study have shown the benefits of such a common mathematical and modelling endeavour [12]. First it would provide a qualitative and/or quantitative framework for diversified landscape modelling; second, a description of landscapes might lead to a synthetic (i.e. systemic or integrative) understanding of the interplay between the various processes involved in landscape dynamics; third, mathematical modelling assists in the analysis of the poorly understood landscape complexity (in the sense of numerous, non-linear and emergent properties); and fourth, the use of such mathematical analysis highlights specific areas of lack of knowledge and encourages further empirical research. With such ambitious hopes, the first objective of this study was to develop analytical and modelling frameworks to express the various rules driving the changes of a complex patchy landscape. Our framework is largely based on the general notion of *Rewriting Systems* (RS) [13].

In a Rewriting System, the objects to be modelled are usually represented as strings of “terms”. Each term itself represents a subpart (or component) of the corresponding object. Rules define how terms may change (i.e. how terms may be replaced by or rewritten as other terms). This principle is very general and has been used in many areas where the natural topology of the objects could be described as a string in an ordered sequence of terms. It has been used, for instance, to automate proving of theorems in systems of deductive logic, to model language grammars, and to simulate the development of various biological and ecological systems. A well-known example of such an approach in environmental sciences is the one that has been developed with remarkable success for modelling plant growth and is known as *L-systems* [14], [15], [16], [17], [18].

Despite its obvious successes, conventional RS suffers from a limitation in the form of its dependence on the use of a string topology. While branching systems, such as sentences of a language or plant growth functioning, can be addressed easily, encoded as they are as strings [14], [16], one has to come to terms with the two-dimensional nature of that of patchy landscapes, a fact that precludes the possibility of handling them as a string. In L-systems, for example, plant branching structures are described as bracketed strings of terms (also called modules) representing the different organs of the plant (shoots, leaves, flowers, buds, root segments, etc.). Declarative rules describe how the different modules change throughout time. Given an initial string representing the plant at some initial date, the rules applied to every term of the string make it possible to compute the next stage of the plant growth. By proceeding iteratively on the successively obtained strings, it is possible to carry out simulations of plant development over several growth periods [14], [16].

If the approach nicely applies to plant branching structures, many other natural systems have a more complex topology (e.g. a cell population in a tissue [19], a network of adjacent landscape units [5], the vascular network of a leaf, etc. [20]). In landscape modelling, the topology of a two-dimensional patch is a planar graph, which does not allow encoding as a string. The requirement that the topology of the object should be a string is thus clearly a limitation of the L-systems approach. To alleviate this constraint, extensions of RS have been proposed to take into account more complex topological structures, in particular in the domain of biology [21], [22]. Extended RS able to account for more general topological organisations, such as graph RS [23] and MGS [21], are currently being developed. However, such systems are based on a topological graph that is more 1D than 2D or 3D, thereby suggesting a need to redefine the associated rewriting rules. For example, such systems have been used to simulate the apical meristem growth of any plant, which is constituted of a set of interacting cells located in the 2 or 3D space [19]. Although not really growing, a patchy landscape is highly similar in terms of modelling to a cell tissue in which landscape patches may represent the cells and land cover neighbourhoods may represent the chemical interactions [5], [19]. These systems are intended to provide efficient frameworks to model dynamic systems whose *n*-dimensional structures change throughout time, and systems that are so frequent in biology and ecology.

In this paper, we hypothesize that the RS formalisms are well adapted to the modelling of landscape dynamics, and we propose a preliminary formalization of this questioning. Our main assumption is that every landscape dynamics of every description, such as patchy and anthropogenic ones [4], [5], may be modelled by a combination of relatively local changes corresponding to module changes. Modules here refer to landscape units (an agricultural patch, a hedge of hedgerow network, a building…) that may be interacting with others at different spatial and temporal scales. This assumption is an approximation of more complicated factor associations, as proposed decades ago by landscape ecologists [2], [3]. We capture the complexity of a multi-component mosaic by dividing modules into types. All modules of the same type share the same description, i.e. they behave according to the same rules, irrespective of the number of occurrences of a given module type within the whole landscape. This makes it possible to keep model specifications concise, even if the simulation eventually were to yield extensive structures that were made up of a large number of modules. Finally, we differentiate in this work approaches to handling patches whose topology changes over time from those whose topology does not. Changing topology is simulated here in a restricted way, in the sense that only patch mergers are investigated, i.e. two patches are transformed into one new patch. In addition, we take into account the hierarchical organization of landscapes at different levels, as unit changes often depend on rules interconnecting successive organization levels (e.g. villages are composed of farmers that are dividing their farms into several units). All of this work has been tested and developed in the DYPAL (for Dynamic PAtchy Landscape [4], [5]) landscape modelling platform, which is the prototype of a free and opensource software written in Java® under L-GPL licence.

Our second objective was to demonstrate the interest and applicability of a RS framework to a model landscape through a simple yet relevant application: we selected a range of grassland managements and simulated them in a French agricultural landscape in order to assess their respective impacts on biological conservation [24]. Although simple, these landscape dynamics undergo non-stationary spatiotemporal changes that still remain a challenge to be modelled, but are rarely explored in the literature. We chose one random plus three realistic rotation systems (i.e. land cover changes) based on existing farm production systems: intensive dairy/beef livestock production, extensive dairy/beef livestock production, and hog (breeding) production. We focused on grassland pattern dynamics because they are of great importance for biodiversity in such agricultural landscapes [25], [26], [27]. In order to sustainably manage the landscape biodiversity, we assumed that a higher grassland frequency as well as a higher connectivity (heterogeneity contagion) was favourable to species conservation. With these assumptions, the central question we aimed at answering was: Which agricultural production system has the lowest impact on biodiversity?

The structure of this paper is as follows: In the modelling section, we first detail the landscape modelling principles, describe the formal model built on the basis of RS, and then explain the key processes involved in landscape changes. In the material section, we list the simulated grassland management scenarios and the chosen study site, and describe the analytical tools used to quantify the simulation results. We finally leave it to the reader to evaluate whether, and how, on the basis of our illustrations and our discussions, an encoding of landscape processes as rules seems a scientifically valid step.

## Landscape Modelling

### 1. Landscape Modelling Using Rewriting Systems

Rewriting Systems (RS) are very close to the notion of the generative grammar introduced by Chomsky to describe the syntax of natural languages [13]. The system consists of a finite set of modules (or components associated with symbols), possibly connected by topological relationships, and of rules of production (or rewriting rules) that define how modules change throughout time. In general, a production replaces a symbol by zero, or one or several new symbols. Finally, RS need a starting axiom which is the initial state of the system modelled. They may represent words in a sentence, as in the original interpretation by Chomsky, but they also may represent organs in a plant, cells in a tissue, landscape units in a landscape, or other components depending on the object studied. The use of related formalisms in the description of such apparently disparate notions as languages and ecological structures may seem surprising at first. Yet, it reflects the common finite and dynamic nature of sentences under construction and developing organisms [18].

**Figure 1:**
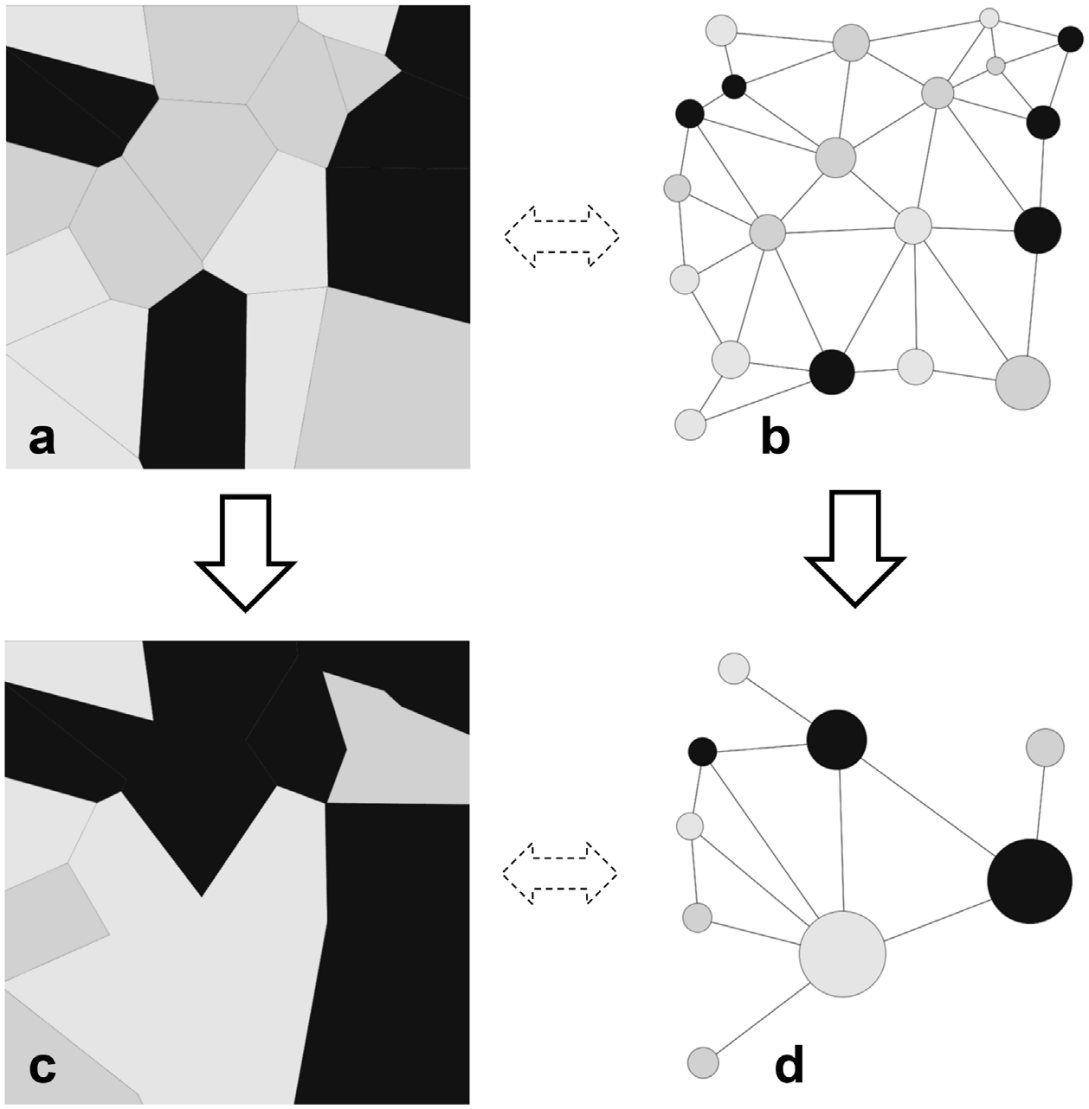
A patchy landscape sequence with its changing topology. A simple patchy landscape viewed by its patch mosaic (a) and its topological graph (b) at time step t, and at time step t+1 (c and d). The node properties displayed in the graph are the main patch attribute (the land cover, in gray scale), and the patch surface (the disc size). Vertical arrows represent time changes of the landscape (1a, 1c) and dashed horizontal arrows represent the link between geometrical mosaics and topological graphs (1b, 1d). Here, merges and random (land cover) rotations, both with a probability p = 0.5, have been applied between the two time steps.

In our study, the landscape at a date *t* is represented by a (planar) graph (Fig. 1a, 1b). This graph is made up of nodes representing the landscape units and edges that connect two nodes if their landscape units are adjacent. These edges are labelled as ‘<’. The starting axiom in the case of a landscape grammar is the initial state of the modelled landscape that is the initial planar graph assembling the connected landscape units. A landscape graph changes over time according to local rules that apply to the graph nodes, thus resulting in the landscape representation at date *t* +1 (Fig. 1c, 1d). These graph nodes correspond to the RS modules, and these rules describe the changes in the different types of landscape units. Additionally, a set of farms is defined at another organization level, and each node of the landscape graph is attached to a farm and its associated farmstead, with relations labelled ‘/’, so that all the units of a given farm make up a connected sub-graph of the initial graph. We usually label as ‘horizontal relationships’ those connected by the neighbouring graph at the same organization level, and as ‘vertical relationships’ the relationships connecting two graphs of different organization levels. Farms are represented with module *F(e)* with *e* being its production system. In the different scenarios considered, farms are visualized as implementing a given production system *e* that suggests a specific land cover proportion within the farm. It has been shown elsewhere that the main factor controlling unit rotation (changes of a landscape element) is this production system and its distance to the farmstead [4]. Unit and farm modules are accurately located, although cartographic coordinates are omitted in the equations for the sake of clarity. In particular, the distance *d* of a landscape unit to its farm is used as a parameter in landscape modules. In this representation, landscape units are specified as modules *M(l,t,a,d).* Hence, we define the neighbourhood of *M* as being the neighbourhood of the corresponding node in the landscape graph. Here a unit is characterized by four variables (this list is determined by the modeller and can be adapted to the case of more variables): *l* represents the land cover type (by definition equal to 1, 2 or 3 for wheat, grassland or maize, respectively), *t* is the amount of time this unit has been set with the land cover *l*, *a* is the geometrical representation of the module (here a polygon), and *d* its distance to the farmstead.

In our landscape modelling, each elementary change in the attribute or in the geometry of a landscape unit is described by a rewriting rule. Rules are described using a specific syntax (see below). Each rule may take into account some parameters related to the unit that should change, or to some module relationships such as farm ownership. In L-systems grammars, the graph is a string of modules. The strategy in those grammars to compute the string at the next time step consists in considering each string module in the order in which it appears in the string, and then looking for a rule that would apply to that module. In our approach, the rewriting strategy is different in that landscape rules are considered first, and for each rule we look for all the units that may be transformed by that rule in the landscape graph, and subsequently apply the rule to them before considering the next rule. However, these two approaches do not lead to similar results. Modules are here processed sequentially within each simulation iteration, and thus the production rules are not supposed to be parallel. This way of proceeding has been suggested by the way patchy landscapes are usually driven, and the rule-centred approach has been shown to be almost always more efficient than a unit-centred approach (see discussion).

The following has been the general strategy for the application of the rules. At each step of the simulation, the derivation algorithm proceeds in successive selections of rules, landscape units, and neighbouring units. A ‘selection’ stage means that not all objects need be used. This leads to the following stages: (i) A sequential selection of the rules is carried out as ordered by the user within a list; (i.1) If the rule applies, a selection is made of a node *n* that bears a module *M(l,t,a,d)* in the current landscape graph. Each node might be selected systematically, or at random, depending on the type of module this rule applies to, or on the proportion of modules to be considered. A specified node might be selected at a time step as many times as there are rules associated with it; (i.2) In the case of a rule needing information on neighbourhood links (horizontal) or other organization-level links (vertical), either a node *M’(l’,t’,a’,d’)* is selected in the neighbourhood of M, or alternatively the farm *F(e)* to which that node belongs is chosen. These neighbourhood selections might be either random or be formalized into more complicated rules, but the context cannot be an already transformed module. The module *M(l,t,a,d)* is then replaced by the right-hand side of the rule. The subsequent selections of *M′* and/or *F* may or may not be random. (ii) Continue with step (i.1) until all the rules have been tried for the current iteration. The simulation stops when all iteration steps have been computed by this algorithm.

### 2. Landscape Rewriting Rules

Rewriting rules (denoted by →, see Table 1 for the full syntax) are defined as follows:
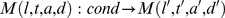
(1)


**Table 1:** The landscape language syntax.

Syntax	Significations and details
*	Unit merger
÷	Unit split or division
+	Unit dilation
−	Unit erosion
.	Unit disappearance
ο	Unit appearance
#	Unit attribute change
[ ] ex. [+ − −]	Combination operation
! ex. !#	Forbidden operation
M(l,t,a,d…)	Landscape unit module and its attributes
:	Definition of the conditions required to apply the rule
/	Spatial context required for the unit operation (left hand side of the rule only)
→	Definition of the rule to be applied on unit
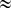	Topological relationship (between similar units)
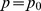	Probability to apply the rule
&	Logical “and”
|	Logical and inclusive “or”
else { }	Condition
< > + −/x …	Usual arithmetic operations (right hand side of the rule only)
S_i_(e)	Surface proportion of production e and unit land cover i

Here is the proposed syntax used in this paper and describing the rewriting rules to be applied on landscape units. It is an exhaustive view of symbols used in this work on landscape dynamics with the DYPAL landscape modelling platform.

The left-hand module *M* is the module that should be replaced. After the colon, a predicate *cond* specifies that the rule can be applied only if *cond* is true. Conditions may be combined using the usual logical operators (‘&’, ‘|’, ‘*else’,* and ‘{}’ in the case of several conditions). The right-hand side specifies the module that replaces the left-hand side module. In this case, exponents are used to differentiate parameters of similar type, on the basis of an algebraic combination of existing parameters and constants of the module. As we shall see in a moment, special functions are allowed in the case of more complicated module manipulations. The new module *M* inherits the topological relationships of its parent module and its attributes (when they have not changed). Stochastic applications of the rule are also possible and will be expressed with the pattern:
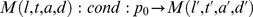
(2)


Here, the rule is applied only if a random test exceeds the proposed threshold probability *p_0_*.

A rule may depend on a context, *e.g.* on neighbouring modules:
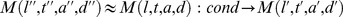
(3)


In this case, the left-hand side is matched only if the following conditions are met: (i) *M* is matched; (ii) the chosen neighbouring module *M* during rule application is matched. Note that, in the absence of additional information, any neighbour may be considered. The model provides a topological (*i.e.* neighbouring) graph for every landscape element category (units, farms, etc.), in which each unit is modelled by a node and each neighbourhood relationship is modelled by an edge. This makes it possible to adapt application of the rule(s) to the specific local context of the module. Rules may possibly rewrite two modules at the same time:
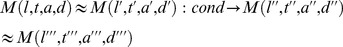
(4)


It is crucial to notice here that in our system, the notion of context is more general than that defined for L-Systems, since we are considering general graphs instead of strings. Moreover, in addition to the notion of context based on spatial adjacency of units, we introduce a notion of context based on the belonging to a farm (or to any other unit belonging to another organization level of the landscape). Such a context is symbolized by the notation ‘/’ giving rules of the following shape:
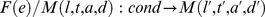
(5)


Agricultural policies, the driving-rules used by farmers and landscape stakeholders, are often expressed according to land cover proportions at the farm or the landscape scale, in a way that every production system *e* defines the crop rotation system of the associated farm. We need the operator 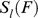 that gives the required proportion of land cover *l* for the production system *e* to which the specific farm *F(e)* and module belong. Such proportions are given as a percentage of the farm surface and are supposed to be approximately reached (variations of some small percentage points are allowed, hard-coded to 5% here). It then becomes possible to adapt applications of the rules to the states of the land cover pre-existing at each step:
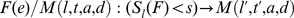
(6)


In this case, the rule applies at the condition that the first land cover (*l = *1 by definition) does not exceed *s* % of the farm surface the unit *M* belongs to.

Interestingly, models that can be described with our system can either be Markovian or not. This simply depends on the rules that the modeller defines. As most rural landscapes would behave in a non-Markovian way, our formalism can occasionally manage sharp changes not related to the previous landscape state. Indeed, most agricultural policy at a definite date is non-Markovian in essence.

### 3. Modelling Key Processes

Landscape units are characterized by a property that represents their spatial configuration (their shape and spatial arrangement). To manipulate this attribute and modify it to model its dynamics, a specific operation has been defined. A typical rule that specifies the merging of two units can thus be defined in the following way:
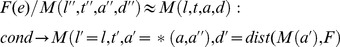
(7)


The new distance between the *M* unit and its farmstead, defined here between their respective centres of gravity, has to be re-evaluated according to the merge function *, and the new polygon shape *a′*.

As our RS formalism is a rule-centred formalism, we have to order the previously identified operations. In the case of pure unit rotations, the driving rule articulation may conveniently be synthesized into an automaton, describing which rotations have to be applied to each landscape unit, depending on their land cover attribute and possibly others [4], [24]. Such an automaton is a graph whose nodes are possible states of the concerned landscape unit and edges authorized transitions between them. Hence, the automaton will not convey information about the land cover proportions imposed by the associated production system the unit is depending on. There are several methods available to succeed in optimally allocating the expected land cover to every unit of the landscape, but they fall outside the scope of this paper and will not therefore be discussed here. In this study, we combined the driving rules of landscape scenarios in an empirical way, on the basis of the modeller’s knowledge. We ascertained in another study that this proposed allocation order is equivalent to the optimum allocation when each farm of the landscape is itself composed of a large number of units.

## Materials and Methodology

### 1. Study Site and Production Systems

Experiments were conducted on a small study site (10.9 km^2^) located in north-western France (Brittany) where agriculture is very intensive. This watershed, the Lestolet, suffers from significant water quality degradation caused by nitrogen present in fertilizer, removal of hedgerows, and abandonment of riparian wetlands, even though the Lestolet exhibits a dense network of hedgerows that has survived with relatively small fields [24]. The European Water Directive asks the authorities and managers to implement a sustainable water management plan that would directly benefit the rich biodiversity of this site, with its location in a humid zone and its remoteness from urban pollution. The Lestolet’s current patchy landscape has been shaped by the manner of land use and changes in land cover over the past three decades, and by the effects of the evolution of agriculture after World War II. The present state of land cover is mostly dependent on the system of production adopted by farmers. Changes in land cover at the farm level come about from agricultural practices such as crop rotation. On a broader scale, such changes can be traced to the spatial distribution of farms and their adaptation to economic constraints and policies.

### 2. Grassland Management Scenarios

To apply our RS formalism to patchy landscape modelling, six scenarios (or simulations) were run on the study site made up of 789 landscape units (fields). A first reference simulation (production system, symbolized by *e  =  A*) was performed with randomly chosen crop rotations. Three other simulations were based on the assumption that all farms of the study site were characterized by a uniform system of production *e  =  (B, C, D)* and its associated land use. That is, to clarify our simulations, we considered every farm of the landscape to belong to the same production system. Scenario *B* was an intensive dairy and beef livestock production, for which the cattle feed was based on maize and hay (temporary grassland), produced on the farms and supplemented with a cash crop (wheat). Scenario *C* was a dairy and beef livestock production managed in an “extensive” way, essentially based on grassland to feed the cattle and for pastures. Scenario D was hog production with a cash crop (wheat). Revenues from the cash crop, wheat, paid for the fodder. For each scenario, landscape changes driven by non-random allocations were already established in previous studies (Gaucherel et al. 2010, Houet et al. 2010). Two other landscape scenarios were performed to demonstrate the degree of relevance of the RS approach. However, these slightly less-realistic simulations, in the sense that their associated operations rarely occur and are never found independently, will not be compared to the four previous scenarios from an agricultural point of view.

**Figure 2:**
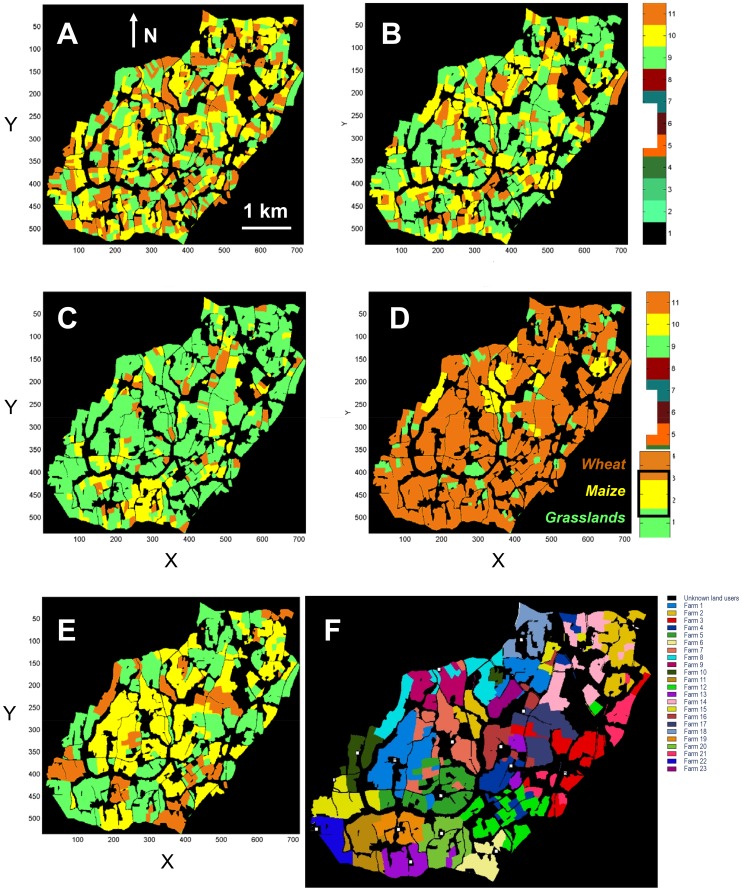
Landscape states of various scenarios. Examples of landscape scenarios for Lestolet crop successions at the hundredth iteration of each simulation with: random (*A*), intensive dairy and beef livestock production (*B*), extensive dairy and beef livestock production (*C*), hog production (*D*) systems and context-dependent random rotations (*E*). Wheat (brown), Maize (yellow) and temporary Grassland (green) land covers are highlighted on a (black) unchanged background. Overview 23 farms of the Lestolet basin, located in cartographic coordinates and using a colour scale (F).

The study site was composed of 23 farms (Fig. 2F) made up of three possible land covers *l  = * (1, 2, 3) * =  (wheat, grassland, maize)*. Maximal land cover proportions 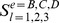, at the farm scale, were quantified by remotely sensed data (SPOT HRVIR and aerial photographs) and *in situ* measurements and interviews. Surface triplets were equal to: 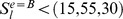, 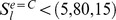 and 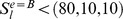 for the three production systems respectively. At the same time, these constraints were not systematically applied in simulations, as land cover proportions used here were averaged estimations with a ±5% margin of error based on more than 130 farms in Brittany.

Two other landscape simulations were performed to add context-dependent scenarios, either with or without topological changes (with a similar proviso for modified horizontal relationships). In order to ensure that islets of crops (maize or wheat) did not appear across the landscape, the fifth simulation (symbolized by *e  = * E) randomly changed land cover if a field had at least one grassland in its neighbourhood, or a change in grasslands otherwise. The sixth simulation (*e  =  G*, the label *F* having already been assigned to the farm *F*) was based on the previous one, and combined with a tendency towards field enlargement. Approximately ten mergers between adjacent fields were observed in the Lestolet landscape from 1997 to 2007 [28]. Such agricultural practice occurs only within a farm and more specifically in land islets [29], but their location is often quite unpredictable. Hence, a field in this simulation was randomly chosen and merged with a randomly chosen neighbouring field owned or used by the same farmer. Fields of areas higher than ten hectares were not changed any further to keep the mergers realistic. It should be pointed out here that the following scenario is not modelled separately as it was already included in the G scenario: landscape dynamics with geometrical and/or topological unit changes (such as mergers), but without context-dependence.

### 3. The DYPAL Modelling Platform

We have incorporated our RS methodology in the *DYPAL* modelling platform (named *L1* in previous studies) to conveniently simulate landscape dynamics. The *DYPAL* platform had several objectives, and combined many of the qualities mentioned in discussions on landscape modelling [5], [8]. We shall briefly state in this section the aims of the *DYPAL* platform [4]: (i) The platform should share common tools and methods dedicated to patchy landscapes; (ii) it should contribute to the design of distinct models and help in carrying out a comparison of their results; (iii) it should be able to transfer models to a wide range of potential users, and to serve as a source of teaching material. With these objectives in mind, the model was developed with a modular architecture and an object-oriented approach, and in an open-source spirit. The kernel around which it was designed provides a stable organizational data structure (spatial representation in vector mode, time-step management, a landscape grammar of our RS formalism and simulated scenarios), and a generic landscape description, which follows.

Each model being developed with the *DYPAL* platform is a set of modules extending this landscape description with a proper data structure and one or more specific changing functions (cultural succession, mosaic fragmentation, forest colonization …). These models or applications have been developed to address a specific landscape issue [30], and have often worked with one or more identified sites. *DYPAL* also provides extensions (viewers and data extractors to check the landscape states), libraries of calculation that can be used by every modeller, and pilots (both batch and interactive) to control the simulation execution. There are many other algorithms associated with DYPAL that will not be detailed in this paper, such as the already mentioned optimization of land cover allocation within a territory. The DYPAL platform will soon be available online upon request.

### 4. Assessment of Landscape Scenarios

Five groups of measures were selected to estimate the averaged statistical landscape property variations [31]. Grasslands and their connectivity in space at landscape scale appeared indeed to play a crucial role in preserving the fauna and flora diversity [25]. We compared the grassland presence frequency between production systems. The mean grassland frequency distribution is the number of grassland allocations of each agricultural unit, divided by the simulation duration (hundred iterations, corresponding to a hundred years), and gathered into fifty classes. The mean grassland surface area was simultaneously computed. Additionally, heterogeneity of the landscape appears to be a generic indicator of the grassland network and its neighbourhood [2], [32], [33].

We estimated three commonly used types of heterogeneities to capture the various landscape configurations to which species could be sensitive [33]. We computed the overall connectivity (heterogeneity contagion), which synthesized averaged connections between the three land uses over the whole landscape. We were interested in the global landscape structure, rather than in a specific field’s response. We derived a second heterogeneity index (still contagion) by only considering grassland-to-grassland connections to quantify the corridor network in the landscape. Finally, a diversity index (evenness heterogeneity) was computed between two landscape classes (grassland and others). Every heterogeneity index was computed precisely within the landscape contours, ignoring the background, and not on the basis of the total rectangular image [33]. A specific *open-source* software (called *MHM*) has been developed for these spatial analyses and is freely available at the author web pages. Uncertainties have been computed for the three heterogeneity indices based on the standard deviation over one hundred iterations (considering that stabilization is either very short or without impact on the index averages). While index curves are shown for peculiar samples, the final compilation of landscape pattern indices is the result of an averaging stage over one hundred similar (Monte Carlo) simulations.

## Results

### 1. Landscape Equations

**Figure 3:**
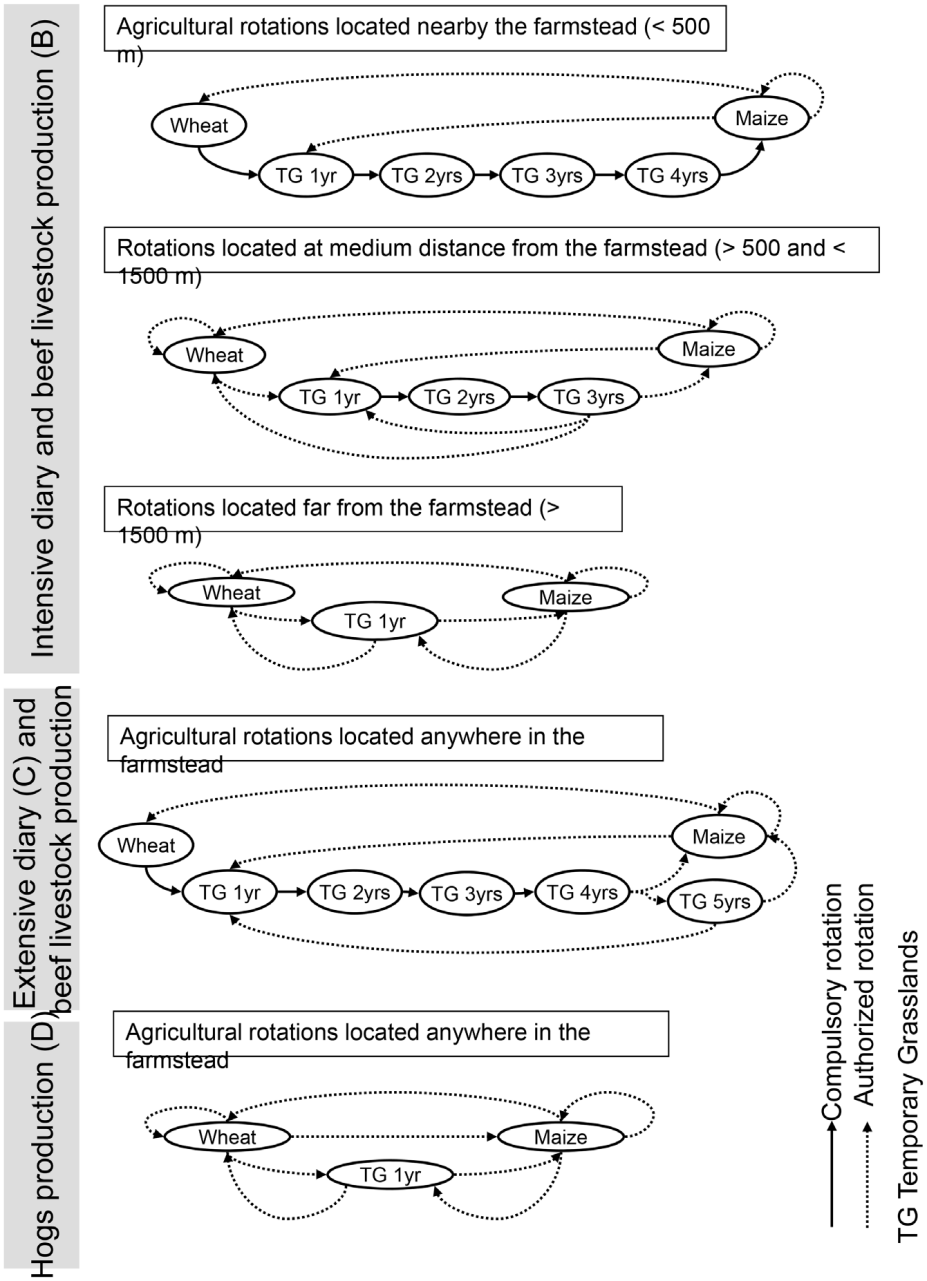
Landscape driving rules of various scenarios. Lestolet site crop successions for inventoried systems of production, namely: intensive dairy and beef livestock production (up, *B*), extensive dairy and beef livestock production (middle, *C*), and hog production (bottom, *D*) systems. Compulsory and authorized rotations (changes) are schematized according to the distance of the farmsteads and to the ages of the Temporary Grasslands (TG).

Given the chosen grammar and the proposed methodology, the model in itself and its formal equations already are a result. Landscape changes driven by random and non-random allocations were established (Fig. 3). In the case of the absence of a production system (scenario *e  =  A*), every landscape unit had to change its land cover into a randomly chosen one:
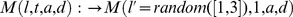
(9)


The *random* function is used and picks randomly one value from the list of values (here three land covers) given as a parameter.

We established the RS implementation and concrete parameterization of equations for scenario *B* (production system *e  =  B*, Fig. 3). Its principle was to express every automaton edge (linking two land cover nodes) by a single rule. The first rotation system, corresponding to units nearby the farmstead (*d* <0.5 km), can be expressed with the following equations:
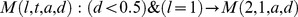
(10)

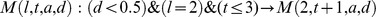


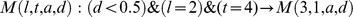





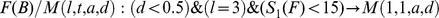


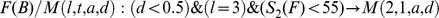



These equations have to be interpreted as such. The first rewriting rule concerns all landscape units with land covers *l = *1 (i.e. wheat), transformed into a unit with the second land cover (temporary grasslands). The *l = *1 statement is a condition of the operation, therefore written before the arrow, while the result itself of the operation (here *l = *2) is written after the arrow. The second rewriting rule, concerning the second land cover, also requires that the unit’s age increases by one (year) at each new time step. The fourth and fifth rules, concerning the third land cover, also limit land cover changes to the specific 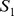 and 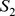 surface proportions (for land covers 1 and 2) of the farm *F* having the production system *B*.

Rules have to be applied sequentially, in the listed order. The following rules concern operations for which the model does not enjoy a large range of change possibilities, due to the already-present land covers (for example, only maize (*l = *3) and wheat (*l = *1) can be exchanged one with the other, and so, this becomes more of a constraining rule).

The second rotation system of this production system *B* is expressed with:
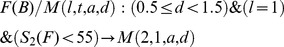
(11)

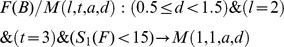


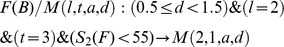


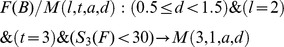


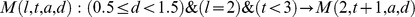


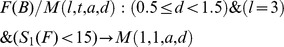


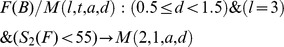



The third rotation system for this scenario *B* is:
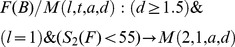
(12)

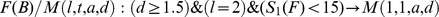


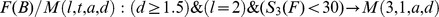


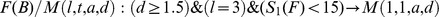


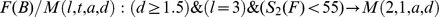



The following two production systems (*e  =  C* and *e  =  D*) do not take into account the farmstead distances *d* (Fig. 3), and this requirement will therefore be removed from the equations. Similarly, the maximal duration of a unit within the same land cover never exceeds unity (i.e. they systematically change), so the age *a* will be removed too. We then have:
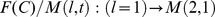
(13)

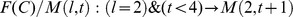


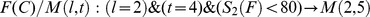


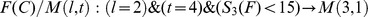


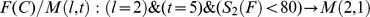


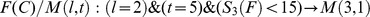


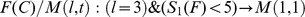


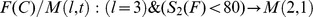



In scenario *D*, patch land covers may change in other ones, depending on the specific surfaces imposed by the production system and for a specific distance to the farmstead (*d* ≥1.5 km):
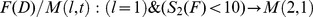
(14)

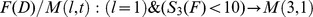


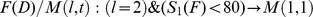


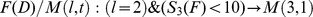


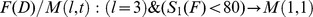


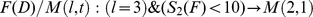



In scenario *E*, every land cover change takes place according to grassland neighbourhood only. Following equation (3), we have:
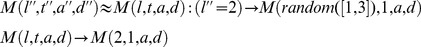
(15)


In this case, the two rules are applied in the same iteration. For each module, rules are sequentially tested within each iteration. If the first one fails, the second is applied (so, this is not an “else” condition). Note that this is the classical rule of application in L-systems.

In scenario *G*, the merging of two units belonging to the same farm is written following equation (7). Its land cover may rotate in any other land cover:
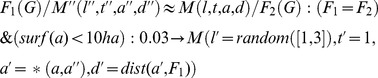
(16)


In this case, we merge two modules belonging to the same farm, if the surface of module *M* is less than 10 ha and with a probability less than the 0.03 threshold. The expression 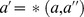 represents the geometrical merger of the two polygonal representations of the units. Insertion of the new module in the topological network representing the landscape is automatically updated. The nodes representing the two units are replaced by a single new node. The edges connecting this new node to the rest of the graph are such that if there was an edge between any of the two parent nodes to a different node of the graph, then there is an edge between this node and the new node. Finally, the rules of scenario *E* are also applied after the merging rule.

### 2. Comparison of Simulated Landscape Changes

**Figure 4:**
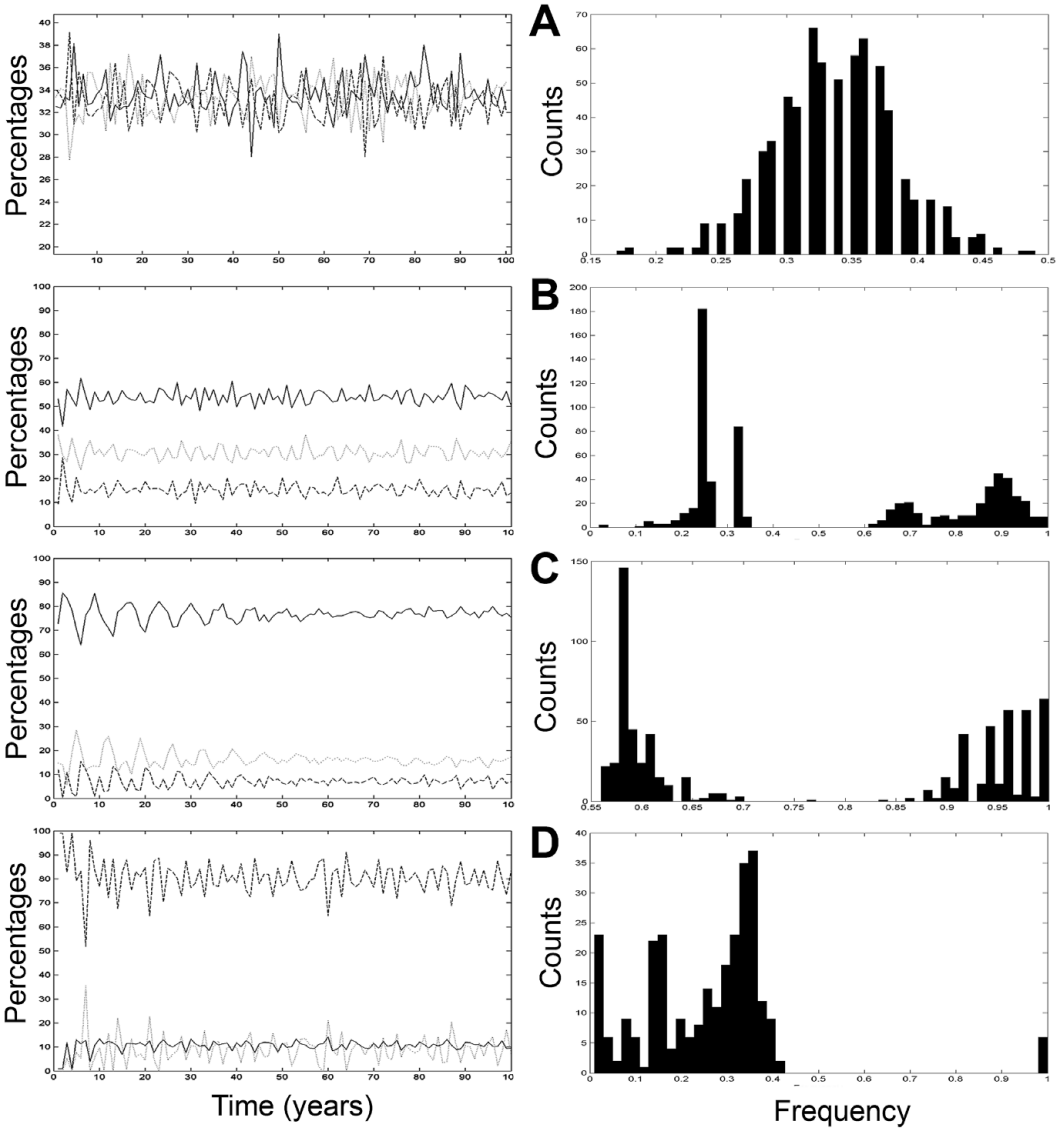
Quantified dynamics of landscape compositions. The three land cover proportions (left-hand side) and grassland appearance frequencies (right-hand side) of the Lestolet during the four hundred-year long landscape scenarios detailed in figure 2. Land cover proportions concern: wheat (dashed line), maize (dotted line) and temporary grassland (plain line)).

Final landscape mosaics showed global as well as local variations between the four scenarios (Fig. 2). For each scenario, we computed the grassland density as well as heterogeneity statistics (Table 2). A first expected result was that randomly generated landscapes (Fig. 2A) differed drastically from the other rule-based scenarios (Fig. 2B, C, D). As simulations were made with landscape unit entities, a rule applied on a specific land cover modified its abundance (proportion) and/or position (spatial distribution) in the landscape. While scenario *A* led to heterogeneous land covers and land uses (25% grasslands), scenario B increased the grassland surfaces (plain lines on Fig. 4B, 55%), with specific positions in the landscape. Scenario *C* increased even more grassland surfaces (Fig. 4C, 78%) even further after a longer stabilization period due to a greater number of compulsory rotations occurring onto all landscape units of the study site. Scenario *D* drastically reduced grassland surfaces (Fig. 4D, 10%). Mean grassland density changes stayed quite stable over the second half of the four simulations, although grassland unit positions and their frequency of appearance showed drastic variations (Fig. 4). Scenario *B* differed from scenario *A* (dominant frequency equal to ∼ 0.33, Table 2) by the addition of some high frequencies (short-term appearances): 0.7 and 0.95. It corresponded to a grassland appearance every 3.05, 1.43 and 1.05 years respectively, estimations being averaged over the 789 landscape units and the hundred-year runs. Scenario *C* not only added high frequencies (Fig. 4C), but also shifted the 0.33 grassland low frequency: grasslands appeared on an average every 1.67 and 1.05 years. Finally, scenario *D* stayed around the random landscape frequency, but with a wide range of appearances (from ∼ 0.4 to 0.01) every 2.5 years to every 100 years.

**Table 2:** Grassland management assessments.

Indices/Simul.	Random (A)	Scenario B	Scenario C	Scenario D
Diversity (OS ×2)	0.337±1.9 10−2	**0.467±1.1 10^−2^**	0.490±1.2 10^−2^	0.102±2.2 10^−2^
Connectivity (OS ×4)	0.671±1.8 10−3	0.597±7.7 10^−3^	0.502±2.4 10^−2^	0.467±5.2 10^−2^
Connectivity (grasslands)	0.121±2.9 10−3	**0.133±2.6 10^−4^**	0.121±3.9 10^−3^	0.063±1.2 10^−3^
Dominant grassland frequencies	0.33	0.25, (0.7), 0.9	**0.6, 0.95**	0.1, 0.3
Grassland areas (%)	33	55	**78**	10
Comments	Reference	Favourable in terms ofheterogeneities	Favourable in terms of grasslandcomposition	Non favourable

Compilation of the five landscape pattern indices for the four landscape scenarios: random (*A*), intensive dairy and beef livestock production (*B*), extensive dairy and beef livestock production (*C*), and hog production (*D*) systems. Indices concern heterogeneity indices (diversity and connectivity) as well as grassland area indices. Favourable index values in terms of grassland management are shown in bold. The last scenarios *E* and *G* are given in the text and not listed here, as they concern rather different landscape managements.

**Figure 5:**
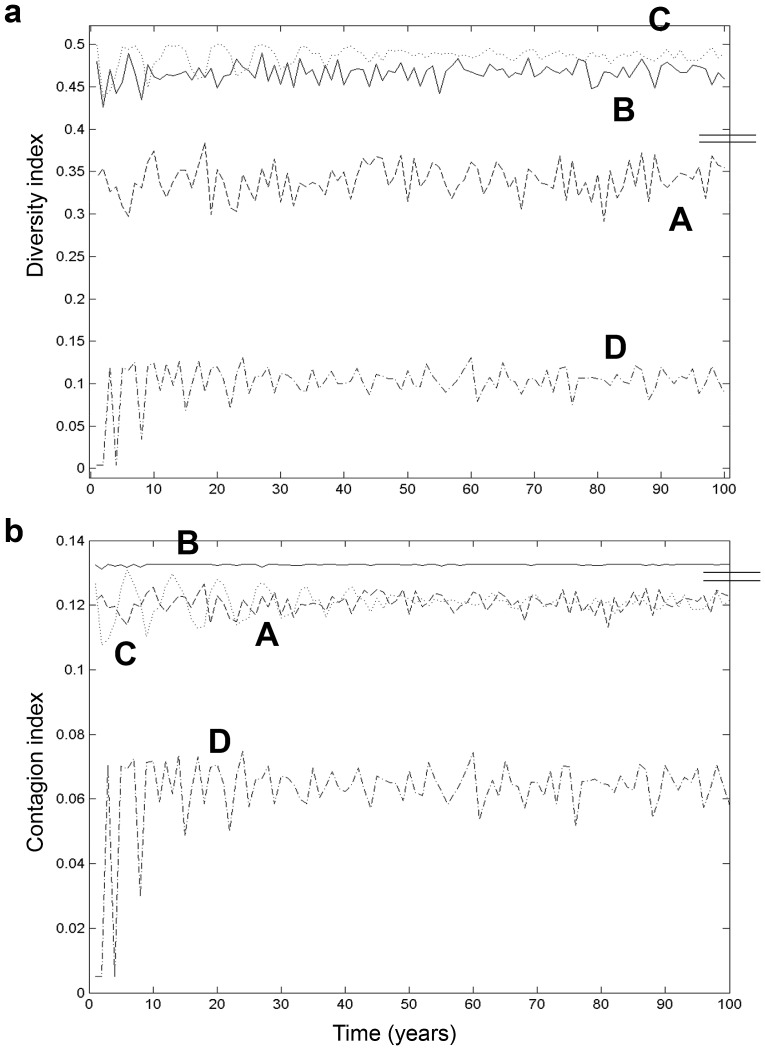
Quantified dynamics of landscape configurations. Diversity (a) and contagion (b) heterogeneity index changes of the four hundred-year long landscape simulations: random (dashed line), intensive dairy and beef livestock production (plain line), extensive dairy and beef livestock production (dotted line), and hog production (dash-dotted line) systems. Small bars at the right hand side of each plot indicate the averaged values of the two context-dependent scenarios (down: context-dependent rotations, up: context-dependent mergers).

While grassland frequency helped in capturing part of landscape dynamics, averaged heterogeneities (and their temporal variations) yielded better information on grassland spatial configurations (Fig. 5). Mean diversity changes revealed quite stationary behaviours between simulations, except the last one reaching a quasi-stable state after almost forty years. Scenarios *B* and *C* showed a statistically higher landscape diversity (∼ 0.46 and 0.49 respectively) than did scenario *A* (∼ 0.34) due to frequent grassland rotations, while lower values were observed for scenario *D* (∼ 0.1) (Fig. 5a). The first two scenario diversities, while still far apart, were quite close to the observed value (∼ 0.71). Grassland-to-grassland connectivity of scenario *C*, after a short burn-in phase (warm-up iterations), was equal to that of scenario *A* (∼ 0.12), while the scenario *B* value was higher (∼ 0.13) and the scenario *D* value much lower (∼ 0.06) (Table 2). Yet, this scenario *B* connectivity was much more stable (i.e. with a lower standard deviation) than scenario *D* connectivity. Finally, the more complete landscape connectivity (Fig. 5b), working with the three land covers plus the background, seemed to broadly follow the same trends, except for the random simulation (*A*): the latter appeared to be closer to scenario *B* than to scenario *C*. Scenario *B* also had connectivity heterogeneities very similar to the observed ones.

The following scenarios (*e  =  E, e  =  G*) were added to highlight some capabilities of the chosen grammar and were correctly simulated by the use of RS (Fig. 1E). They display roughly similar heterogeneity values, for diversity (∼ 0.39±1.4 10^−2^ and 0.40±3.2 10^−2^ respectively) as well as grassland connectivity (∼ 0.130±1.5 10^−3^ and 0.129±2.8 10^−3^ respectively). Grassland frequency of scenario *E* is normally distributed around ∼ 0.42±0.014 (not shown), while a similar index is not available for merged fields of scenario *G*. Although this was not expected, both scenarios finally exhibited approximately 40% grassland areas. This finding could be considered a demonstration of the capability of our landscape language to simulate and to qualify a wide range of landscape dynamics.

## Discussion

In this study, we used *rewriting systems* (RS) to formalize and model patchy landscape changes. This was our first objective. The basic assumption underlying these models was that such landscape patterns are the result of local changes of various modules (the landscape units). Modules of the same nature exhibit varying behaviours depending on their states (i.e. the values of variables that are associated to each module) and depending on the signalling between the modules (Fig. 3). The convenience of expressing context-sensitivity in dynamically changing modules, such as in scenarios *E* and *G*, is an essential property of RS [12], [17].

### 1. Landscape Change Diversity

In this study, we aimed at illustrating most landscape dynamics encountered in agricultural zones. These man-made landscapes are difficult to formalize because they do not have regular dynamics and because, unlike natural landscapes, they relate to complicated and numerous decision rules. Module (landscape unit) changes concern either compositional (land cover) changes or configurational (geometrical and/or topological) changes. Furthermore, unit changes are context-dependent (or context-independent) when they involve the neighbourhood relationships to be updated. Here, we chose to illustrate three of these four classes of landscape unit changes, with an emphasis on the most common one: scenarios *B*, *C*, and *D* concern compositional changes without context dependency. Scenarios *E* and *G* are context-dependent, for either compositional or configurational landscape dynamics. Formalization and simulation stages were successful in modelling such complex dynamics in all cases (Fig. 4). Hence, we are confident that RS would be adequately efficient to model a large panel of landscape dynamics. For example, more complicated rules, adapted to other agricultural, forested or peri-urban landscapes may be conveniently defined and modelled through RS. At the same time, we would also need a less empirical way to set priorities for the rules, a goal outside the scope of this paper.

The main appeal of this work has to be emphasized here. A distinctive feature of RS formalisms is that they give rise to a generic class of programming languages with a potential for use in specifying landscape models. This property of RS made it possible to construct a generic simulation software (here called DYPAL) that possesses the capacity to model a large variety of landscape dynamics. In particular, RS handles both Markovian and non-Markovian dynamics that are commonly found in man-made landscapes [5], [24]. RS is thus able to manage classical Markovian dynamics such as transition probability between land covers or other hidden-Markov-chain approaches [9], [34], as well as more complicated non-linear processes such as sharp vegetation transitions undergoing natural or anthropogenic perturbations. Although it was not our intention here to prove this assertion, it is always possible to add a sharp rule starting at a specific iteration of the simulation.

Hence, RS offers the opportunity to formalize land cover and land use changes that are most of the time modelled without any formalization. The majority of currently known landscape models indeed are inefficient to analyse the coherency and the properties of the changes they allow [6]. Therefore, we need a coherent and self-sustaining approach able to capture a wide range of landscape units (polygons, polylines …) and a high diversity of dynamics (Markovian, non-Markovian …). We should not be intimidated by the apparent differences between the growing structures of plants to which RS have been applied and the stable boundary of a dynamic patchy landscape. Indeed, there is a critical common point between these expanding and non-expanding dynamics: they change their inner structure and the spatial relationships between their constitutive entities. In other words, the intrinsic topology of the structure is rapidly evolving in both cases, and this is what strongly suggests context-dependent formalization to model non-expanding systems [8], [19]. To formalize changes with a formal grammar opens the way to a finer analysis of dynamic properties such as: regularities (invariants), equilibrium if ever (trajectories and asymptotical behaviours), and coherency (conflicts and redundancies). This will be the next stage of this working program.

### 2. Grassland Evolutions

The second objective of this work was to assess whether it is possible to estimate, with the landscape language tool, the risk for some agricultural production systems to favour biodiversity erosion or, at least, to impact some species community. We intended to quantify such possible impacts by the use of the grassland frequency and a set of heterogeneity indices [31]. On the temporal aspects of the simulations, the return time of grasslands and their main frequency seemed more relevant in this study than did the age of the grassland, which simply followed the automaton constraints. We made here the assumption that the more grassland surface, presence frequency and land cover connectivity, the more species have a chance to be preserved [25], [26]. Connections between different crop fields partly regulate biological (and geochemical) fluxes in the landscape, and suggest the need to compute a global landscape structure index based on the three dominant land covers grassland, maize and wheat [2]. Grassland to grassland connections as well are relevant for species, such as small mammals, potentially moving from one grassland patch/unit to another by the way of these landscape corridors [25], [30]. Finally, a diversity (evenness) index was computed between two landscape classes (grassland and others) in the case of some species, such as some bird predators, being sensitive to grassland presence rather than to grassland connections. We chose a wide panel of production systems allowing a fruitful comparison of various grassland managements (Fig. 2 and 3).

A close inspection of an intensive dairy and beef livestock production scenario (scenario *B*) revealed overestimation of grassland surfaces (55%, Table 2). Indeed, scenario *B* was the closest to the real landscape changes for all indices. At least, dominant grassland frequencies were more significant (modes of values 0.25, 0.7 and 0.9, around two times higher and larger) for real dynamics than for scenario *B*. Such frequency modes were caused by farm allocation properties chosen by farmers. They suggest that better elaborated rules (using mixed grassland managements in the same landscape) would improve the scenario’s realism [28]. We remind the reader at this point that the observed landscape is made up of a mosaic of the three production systems, with a large number of intensive dairy and beef livestock production units. Scenario *B* grassland connectivity was also much more stable (i.e. with a lower standard deviation) than it was for other simulations. This observation was a direct consequence of the number of landscape units eligible for inclusion in the respective production system or, in other words, of the number of constraints following the grassland management. Indeed, beef production landscapes (*B*, *C*) were less flexible in making changes in than was hog production landscape (*D*), but still had a high grassland proportion. Finally, the landscape connectivity based on the three land covers seemed to broadly follow the same trends between scenarios, except for the random simulation (*A*).

This important point proved that grassland dynamics did not follow the same logic (i.e. changing mechanisms) as the overall landscape dynamics. Management specificities were then highlighted by these two different connectivity trends: the three land covers together were more homogeneous, relatively to random distributions, than grasslands alone. Our results revealed that dairy and beef livestock production systems (scenarios *B* and *C*) were both more favourable to wild species, but for different reasons: intensive dairy production has high heterogeneities, while extensive dairy production has higher grassland proportions and return frequencies. Hog production was far less favourable to species relatively to our conservation constraints. A combination of both intensive and extensive dairy production systems would probably lead to the most favourable management in terms of biological conservation. While these scenarios were still unrealistic (because they had a unique production system in the landscape), they proved that a the study and modelling of grassland managements can greatly improve biological conservation [27].

Both context-dependent scenarios (context-dependent rotations *E* and context-dependent mergers *G*) exhibited averaged values of heterogeneity: both had higher standard deviations, mainly due to the higher field sizes. In addition, they appeared to be relatively close to the real landscape in terms of heterogeneity, but not in terms of grassland frequencies: this was quite natural for such neighbouring rules, as neighbourhoods have the effect of smoothing spatial variations, but not of systematically smoothing the temporal variations. The 10-ha threshold imposed to more realistically limit merging led to a reduced number of islets within the road network or the farm boundaries. Finally, the landscape language formalized three of the possible landscape dynamics encountered: composition changes with context-dependence (scenario *E*) and without context-dependence (*B*, *C*, *D*), and configuration changes with context-dependence (*G*). We did not model configuration changes without context-dependence, as it was already included in the last scenario (*G*). Also, it is worth pointing out that our work did develop the other possible *generic operations* encountered in patchy landscapes: we used a unit rotation (symbolized by #, see Annex 1) and merge (*), although we are likely to later need a unit split (÷), dilation (+), erosion (−), appearance (.), and disappearance (¤). Most of these rules have the effect of changing the landscape topology as they concern configurational operations. A last generic operation is required in order to forbid a specific operation *x* (denoted ‘!*x*’), not to be confused with ‘no change’ (by default, and expressed by the rule M → ε). This is another fruitful perspective of our working program.

### 3. Towards a landscape language

Emerging landscape patterns are the results of numerous and non-trivial driving rules that are hidden and difficult to infer from the landscape patterns. RS allows one to generate and to characterize them, on the basis of an identified dictionary and of grammatical rules (a syntax) corresponding to the unit properties and to the unit change operations, respectively. This is the first stage to define what we called by analogy a *landscape language*. As in linguistics, an incorrect syntax would alert the user that the corresponding landscape change should not exist (or that a forgotten rule should be defined). Conversely, it becomes easy to define and explore new possible landscape changes, built on the basis of the accepted dictionary and syntax. Yet, the landscape language proposed in this study is only a preliminary attempt to formalize landscape dynamics. The DYPAL language is an adaptation of existing RS to landscape specificities, and will still need to be refined. Indeed, we modified some of the central assumptions of RS to fit our needs related to the patchy object being simulated and to its functioning. Hence, three successive stages led us to the definition of a language and a grammar adapted to the landscape objects.

Firstly, L-systems or similar formalisms based on the rewriting of strings appeared to be irrelevant to an attempt to formalize patchy mosaics that are two-dimensional structures. We drew inspiration from other RS using grammars on graphs and on networks [21], [23]. They suggested convenient specifications for complex topological changes. We still have to find the trade-off between drastic topological and geometrical changes that are not easy to specify, and our wish to keep the formalism as easy as possible for the use of ecologists (in the wide sense). The challenge hidden here is to provide expressivity without losing readability. Finally, the integration of the landscape language described here into the DYPAL modelling platform has to be completed [4]. Up to now, every rewriting rule has had to be defined through a user-friendly interface of the software. We expect to directly implement the landscape language (the coded equations) with a parser into DYPAL, to enable more extensive utilization of the powerful possibilities of a language-based approach.

Secondly, we modified the concept of context-dependency inherent to RS [13], [18], into a multilevel framework. A rewriting rule is context-dependent when it needs to know the state of the local neighbourhood (i.e. it needs to be connected to it) to deduce the change in the concerned module. Agricultural landscapes have dynamics driven by neighbourhoods as well as by large clusters of units, such as regions, villages, farms, or islets [24]. This knowledge suggested the taking account of more global context than nearby (local) units of the changing module. For this reason, we introduced operators aiming to query the global state of the landscape. As an illustration, the DYPAL formalism and its global context-dependent rules have shown how to manage landscape units belonging to a farm [5]. In brief, to focus on local changing rules, such as would occur in usual RS, is not appropriate to model teleonomic landscape changes (i.e. changes driven by a goal or an optimization target). These formalisms are adapted to local and Markovian dynamics centred on modules constituting the modelled object; it is therefore difficult to control them with a more global objective, a priori “hidden” (i.e. unknown) for every landscape unit. For example, most agricultural landscape changes are driven by the economic market or, as we have seen, by some farm and regional decisions pushing the mosaic towards a more or less predictable state [10], [24]. We still have a long way to go towards managing such global objectives in RS.

Thirdly, another way to manage this wish to have a global goal in landscape dynamics is to no more use a unit-centred approach (as in usual RS), but instead than to modify it in a rule-centred approach. For example, L-systems are supposed to go through every system module (the landscape units) and to apply available rules to it. Conversely, it is possible to go through every rule defined for the system’s dynamics and to apply them to every concerned unit. In the case of landscape dynamics, we verified that more faithful rule-centred approaches were always more efficient than unit-centred approaches: this finding is true for a small number of landscape units and random rules (scenario A), and vanishes into roughly equivalent approaches with large numbers of units (not shown). With a large set of landscape units, the system reduces conflicts sometimes arising between rules, as it offers more opportunities to apply every rule. Yet, the main argument for using a rule-centred approach is that it more closely mimics the way farmers and other stakeholders manage their territories. Simulations this study are a good illustration of this assertion (Fig. 4). Yet, it suggests the need to carefully define the way the user should prioritize the driving rules of the modelled landscape. It could be empirical (as in this study), or more objectively defined with the help of an optimal algorithm, and is necessary in any case.

In summary, there are two important differences between the proposed RS formalism and other elaborated landscape models such as multilayer cellular automata. A spatial difference allows RS to manage irregular neighbourhoods, which is a crucial point when we study highly irregular mosaics. A temporal difference concerns the driving rules of the dynamics that are highly non-Markovian (and non-stationary), for example in human-based landscapes [5]. Furthermore, our formalization combines these two critical differences into the same coherent framework. Even more importantly, the approach provides a rigorous mathematical formalization of studied processes, which most cellular automata are not able to provide, except for relatively regular structures. This formalization is relevant for any layer (between patches, linear networks, farms, regions, etc.) built on complex topologies. Finally, these complex topologies are highly dynamical and need an appropriate modelling framework, here borrowed to DS^2^ [14], [20]. While cellular automata always have a fixed relational graph (the regular lattice topology), our language deals with changing topologies and manage these changes as well as their consequences on the landscape unit dynamics. Today, we would not have been able to formalize these complex landscape dynamics by any other means.

### Conclusion

To cite E. Becker and B. Breckling [35]: “To us, it seems more productive to make use of advances in […] formal approaches that are already well-known for the benefit of ecology. This could help in the process of identifying and filtering out coherent elements and patterns from among the mass of details and data”. By way of a ready-to-hand analogy, we could mention the metapopulation equation that quantitatively explained, for the first time, a population dynamics and its consequences [36]. There is a plethora of other examples to demonstrate how our understanding of a seemingly hard-to-grasp scientific phenomenon improved after a mathematical technique was applied to a study of it.

The landscape language may lead to a better understanding of patchy landscape dynamics. We succeeded in this preliminary work to formalize a wide range of multilevel landscape dynamics. Furthermore, we modelled these dynamics on the basis of such DS^2^ formalism able to manage changing topologies. We believe, on the basis of our findings from the research this paper embodies, that a wide variety of natural and anthropogenic forces may be meaningfully modelled by the use of formal grammars. We believe that these developmental algorithms will find a growing use in the future, as they provide insights into ecological landscape processes that are difficult to obtain through observation and quantitative reasoning alone.
